# Cathepsin S as a potential therapeutic target for chronic pain

**DOI:** 10.1016/j.medidd.2020.100047

**Published:** 2020-09

**Authors:** Karli Montague-Cardoso, Marzia Malcangio

**Affiliations:** Wolfson Centre for Age-Related Diseases, Institute of Psychiatry, Psychology and Neuroscience, King's College London, Guy's Campus, London SE1 1UL

## Abstract

Chronic pain is a distressing yet poorly-treated condition that can arise as a result of diseases and injuries to the nervous system. The development of more efficacious therapies for chronic pain is essential and requires advances in our understanding of its underlying mechanisms. Clinical and preclinical evidence has demonstrated that immune responses play a crucial role in chronic pain. The lysosomal cysteine protease cathepsin S (CatS) plays a key role in such immune response. Here we discuss the preclinical evidence for the mechanistic importance of extracellular CatS in chronic pain focussing on studies utilising drugs and other pharmacological tools that target CatS activity. We also consider the use of CatS inhibitors as potential novel antihyperalgesics, highlighting that the route and timing of delivery would need to be tailored to the initial cause of pain in order to ensure the most effective use of such drugs.

## Highlights

• Cathepsin S plays a key extracellular role in the underlying mechanisms of chronic pain
• Pharmacological tools provide crucial evidence for this role and the therapeutic potential of targeting Cathepsin S
• The route of delivery and timing of cathepsin S inhibitor administration should be tailored to specific causes of chronic pain

## 1 Introduction

Chronic pain is a distressing condition that dramatically reduces the quality of life of those afflicted. Whilst acute pain is a necessary process that has a protective function, chronic pain is maladaptive, outlasting the initial trigger of pain. Characterised by spontaneous pain, heightened sensitivity to noxious stimuli (hyperalgesia) or pain in response to innocuous stimuli (allodynia) [1], chronic pain conditions are currently poorly managed in the clinic. Advances in our understanding of the underlying mechanisms, which can guide the development of novel, more-effective analgesics, is therefore greatly needed.

Chronic pain has been historically attributed to ‘neurocentric’ mechanisms, in other words, being ascribed purely to neuronal responses to disease or injury and existing analgesics have been developed in accordance with this. Currently however, both clinical and preclinical studies have enabled us to appreciate a critical role for the immune system and its bi-directional communication with neurons in chronic pain mechanisms [2,3]. Within the immune response, a key role is played by endo-lysosomal proteases. A major class of lysosomal cysteine proteases is represented by the cathepsins. Here we consider the role of one such cathepsin, Cathepsin S, in the underlying mechanisms of chronic pain. We discuss evidence obtained from the use of drugs and other pharmacological tools that disrupt CatS activity, which has highlighted the mechanistic importance of CatS. The evidence considered here also leads us to conclude that the targeting of CatS is a promising and novel approach for the treatment of chronic pain.

## 2 Cathepsin S expression, functions and release

Cat S belongs to the family of papain-like cysteine proteases, which are widely expressed in animals and plants as well as bacteria and viruses [4]. CatS, like most cathepsins, is a small, monomeric endopeptidase [5]. Following its synthesis in the endoplasmic reticulum, CatS is found as pre-proCatS, an inactive zymogen, in the lysosomal compartment [5]. Here CatS becomes active following the removal of the pro-peptide by other proteases, as well as autocatalytic cleavage, which is facilitated by the low pH in the lysosome [6,7]. A major determinant of lysosomal protease activity is pH – with the majority of proteases only being able to function within a relatively narrow pH range. CatS however, is the only cathepsin that retains its enzymatic activity at a neutral pH [7].

Papain-like lysosomal cysteine protease expression can be found in nearly all cell types. The expression pattern of CatS however, is tissue-specific, being exclusively expressed by antigen-presenting cells including dendritic cells [8], B-lymphocytes [9], macrophages [10], and microglia [11,12]. In these cells, intracellular CatS is involved in adaptive immune responses that contribute to the process of antigen presentation. Such responses include the degradation of the MHC II-associated chaperone invariant chain (Li) from MHC II [13] as well as the generation of peptides from foreign proteins [14,15].

Mice that are deficient in CatS have a reduced susceptibility to models that recapitulate autoimmune disorders such as the collagen-induced arthritis (CIA)model for rheumatoid arthritis [16], whilst pharmacological inhibition of CatS using orally-active therapeutic doses of brain-penetrant drugs serves to effectively reduce clinical scores in both the CIA model [17] and the encephalomyelitis (EAE) model of multiple sclerosis [18]. This effect of CatS inhibition has been attributed to a shift in the proportion/differentiation of T-lymphocytes from a Th1/Th17 type to that of a Th2 type [17].

Aside from the intracellular roles that CatS plays in antigen processing and presentation, CatS also possesses roles extracellularly when released from cells such as macrophages and microglia for instance, due to its ability to retain its enzymatic activity at extracellular pH, as mentioned above. Such release is elicited by pro-inflammatory stimulation, for example nerve growth factor and lipopolysaccharide (LPS) [[19], [20], [21]]. One of the initial effects observed downstream of CatS release is the degradation of myelin basic protein as well as extracellular matrix components [19] and in light of this, the role of extracellular CatS in underlying pathology of disease has previously generated a substantial amount of interest [22]. One of the mechanisms that has been found to mediate CatS release has been identified in microglia. Following pro-inflammatory mediator stimulation, release of CatS from microglia has been shown to be dependent on purinergic signalling. Specifically, whilst the stimulation of Toll-like receptor 4 (TLR_4)_ receptors on primary cultured microglia with LPS alone does not result in the release of CatS, when high concentrations of ATP are administered simultaneously to LPS, CatS release is elicited [23]. ATP-activated P_2_X_7_ receptors are expressed by microglia and indeed, pharmacological inhibition of P_2_X_7_ receptors prevents LPS/ATP-induced CatS release from microglia [23]. Pharmacological studies have indicated that both phospholipase C and A_2_, as well as p38 MAPK (mitogen-activated protein kinase) and mobilization of intracellular calcium ions, are critical for ATP-mediated release of CatS from microglia [23].

## 3 CatS and preclinical chronic pain

There is highly convincing clinical and preclinical evidence demonstrating that extracellular CatS plays a crucial mechanistic role in chronic pain. Indeed, elevations in CatS expression and activity have been observed in tissue obtained from patients with chronic pain, in which the heightened activity is attributed to a decrease in cystatin c, which is an endogenous inhibitor for CatS [24]. Preclinically, expression levels are significantly increased in lumbar dorsal root ganglia (DRG) and the dorsal horn of the spinal cord following peripheral nerve surgical injury, as well as at the site of damage itself [10,11,25]. In such models, the elevation in CatS in the DRG and at the site of injury have been attributed, at least in part, to the infiltration of monocytes/macrophages, which are known to express CatS [10], although it is also possible that the proliferation of resident macrophages could also account for increases in CatS [26]. In the spinal cord dorsal horn however, surgical peripheral nerve injury results in the release of CatS from activated microglia – specifically in regions in which injured nerve fibres terminate [11].

Evidence for the pro-nociceptive nature of increased CatS expression, as opposed to changes in expression being an epiphenomenon to injury, has been provided by studies utilizing pharmacological tools to target CatS signalling. Intrathecal delivery of activated recombinant CatS for example, results in a dose-dependent allodynia in naïve rats within 30 min of delivery suggesting that the role of CatS in pain-like behaviour is causative [11]. In the context of preclinical neuropathic pain following peripheral nerve injury, the effects of spinal administration of the irreversible, non-brain penetrant, non-specific inhibitor of CatS; LHVS (morpholinurea-leucine-homophenylalanine-vinyl phenyl sulfone), also indicate that the role of microglial CatS may be more crucial in the maintenance of pain as opposed to the initial induction. For instance, spinal administration of LHVS results in the reversal of established allodynia in neuropathic rats at both 7 and 14 days post-partial sciatic nerve ligation (PNL). This period of time coincides with the maximum level of expression of CatS in the spinal cord. LHVS spinal administration is relatively ineffective at reducing allodynia at 3 days post-PNL however, when spinal CatS is sub-maximal [11]. This data suggests that in this particular model of neuropathic pain the role of microglial CatS specifically in the induction of pain-like behaviour is negligible and instead is more critical for the maintenance of allodynia. Indeed, prolonged treatment with LHVS, which is achieved by continuous delivery via an intrathecal pump, is more effective at reversing allodynia when applied during the second week of the PNL model, when allodynia is established, as opposed to the first week [11]. Moreover, dorsal horn microglial activation ipsilateral to injury is also significantly reduced when LHVS is administered between days 7 and 14 post-PNL, indicating that microglial activation-mediated release of CatS is essential in the maintenance of allodynia in this model. Similar findings that also highlight the importance of spinal CatS have also been obtained from studies in which inflammatory pain models have been utilised. In the rat CIA rheumatoid arthritis model for example, prolonged intrathecal delivery of LHVS attenuates allodynia as well as the spinal microglial response [23,27]. The use of alternative pharmacological tools has also provided support for the crucial role that CatS plays in the maintenance of neuropathic pain. Inhibition of CatS by intraperitoneal delivery of Z-Phe-Leu-COCHO (Z-FL), which cannot cross the blood brain barrier, attenuates both established allodynia as well as microglial proliferation when given 14 days post-L4 nerve transection, but is not effective when given during pain induction [28].

Whilst such pharmacological tools are critical for enabling us to establish the importance of CatS in the underlying mechanisms of pain, in terms of the translatable effectiveness of CatS inhibition, evidence from drugs that can be used clinically, is also essential. In the last fifteen years or so, dozens of small molecule CatS inhibitors have been developed, which vary in structure and binding strategies [29]. In more recent years, the development of small molecule inhibitors that target other cathepsins, such as CatK, which plays a more crucial role in conditions such as osteoporosis as opposed to pain [30], has received more interest. However, clinically-viable CatS inhibitors, which show specificity for CatS over other cathepsins [31], have still provided us with crucial information regarding the therapeutic potential of targeting CatS in the context of chronic pain. Indeed, inhibition of CatS, using orally-active, centrally-penetrant selective inhibitors exerts dose-dependent anti-allodynic effects in the PNL model of neuropathic pain [32,33]. Whilst the use of orally-active, centrally-penetrant inhibitors of CatS alone results in a substantial reduction in preclinical nerve injury pain, it also further improves the anti-allodynic effects of pre-existing therapies such as Gabapentin and Pregabalin [33]. Targeting CatS centrally could therefore constitute of novel component of combination therapy for the treatment of chronic pain. Whilst the effect of such drugs can be attributed to central mechanisms, the effect of inhibition of peripheral CatS should still be considered.

The use of clinically-viable drugs that target CatS has also demonstrated that in certain models of neuropathic pain, CatS also plays a crucial role during pain induction. The time point at which the role of CatS activity is mechanistically most important therefore varies with pain model. Specifically, recent evidence from a preclinical model of chemotherapy-induced neuropathic pain suggests that an increase in CatS in the spinal cord, which in this case is attributed to infiltrating monocytes, is not only observed during the induction of pain-like behaviour, but play an important mechanistic nociceptive role. Specifically, in the vincristine (VCR) model of neuropathic pain, in which allodynia is observed within 24 h of the first chemotherapy dose, oral administration of a centrally-penetrant CatS inhibitor significantly reduces the severity of VCR-induced allodynia in the first 24 h [34]. Importantly, oral administration of a CatS inhibitor that does not penetrate the CNS does not have an appreciable effect on VCR-induced allodynia [34], strongly suggesting that inhibition of CatS in the spinal cord specifically is required for an anti-nociceptive effect to be observed. In the case of the VCR model of neuropathic pain however, the source of elevated CatS in the spinal cord is unlikely to be microglia, as it has been previously established that microglia are not activated in this particular model [35]. Instead, alterations in vascular permeability at the blood-spinal cord barrier enable CatS-expressing circulating monocytes to infiltrate into the spinal cord [34]. Taken together, these data suggest the role of CatS in the spinal cord in preclinical chronic pain varies depending on the initial injury as well as the source of CatS elevation. Should CatS inhibitors be used as novel treatments for chronic pain, it appears that they would be used most effectively if their delivery route and timing were tailored depending on the cause of nerve injury.

## 4 CatS pro-nociceptive cellular mechanisms

### 4.1 Central mechanisms

When considering the precise mechanisms by which CatS could exert pro-nociceptive effects, two possibilities were initially considered. Specifically, CatS activity could either liberate a pronociceptive mediator or it could cleave an anti-nociceptive mediator and thus disrupt its activity. Indeed, we now know that the former possibility accounts, at least in part, for the pro-nociceptive effects of CatS. One protein, which is cleaved by CatS and has a well-established involvement in the underlying mechanisms of chronic pain, is the neuronal chemokine CX_3_CL_1_ (or fractalkine, FKN), which is principally expressed by neurons [36]. FKN exists as two forms: full-length, membrane-bound FKN and soluble FKN (sFKN), which possess distinct functions from each other. Whilst full-length FKN possesses an adhesion function in the vascular system, sFKN serves as a chemoattractant for immune cells, such as monocytes, and is also essential for transendothelial migration of monocytes that express the FKN receptor; CX_3_CR1 [37,38]. The constitutive cleavage of FKN is mediated by the metalloprotease ADAM 10 [39], whilst cleavage under adverse conditions is mediated by ADAM 17 [40] as well as CatS [36]. Whilst FKN is known to possess crucial homeostatic functions, sFKN specifically is pro-nociceptive, with sFKN-mediated activation of CX_3_CR_1_ on microglia or macrophages resulting in the release of pro-nociceptive cytokines [41]. Bearing this in mind, potential therapies that target FKN signalling in the context of pain, would ideally preserve the homeostatic functions of FKN and target sFKN specifically in order to minimise undesirable side-effects. It is therefore perhaps intuitive that targeting the cleavage of FKN, specifically that which occurs under adverse conditions, could provide an effective therapeutic strategy. The cleavage of FKN could therefore account for some of the pro-nociceptive effects of CatS as well as some of the anti-nociceptive effects of CatS inhibition.

**Fig. 1:**
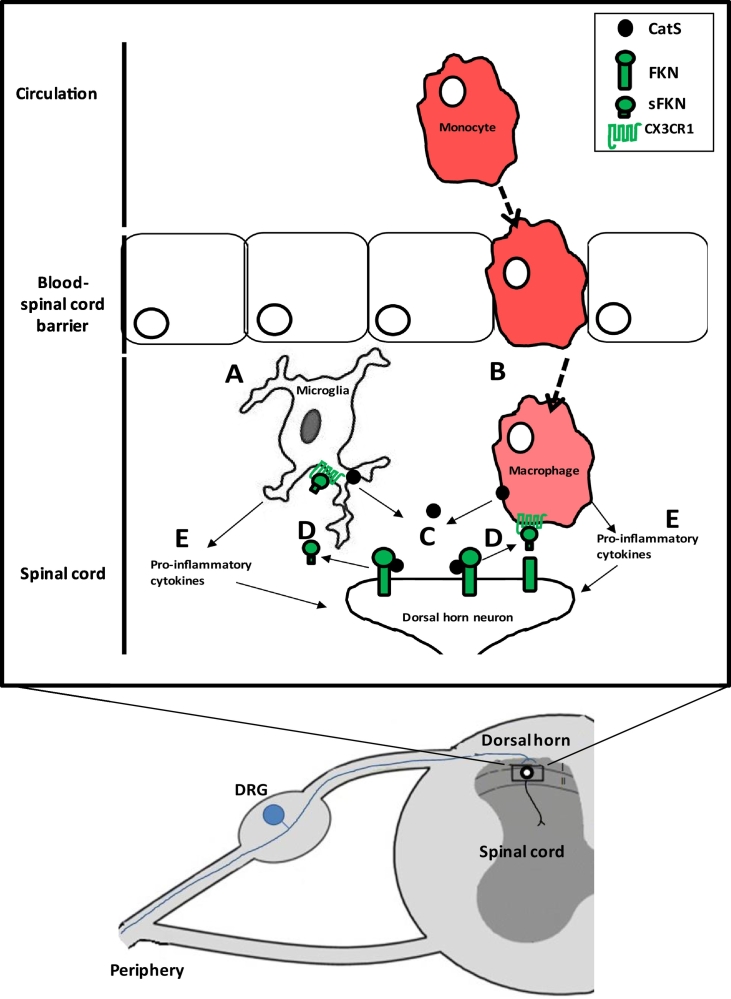
**Potential sources of increased Cathepsin S in the spinal cord in neuropathic pain models.** Depending on the model of neuropathic pain, an increase in CatS in the spinal cord can come from different sources. In the case of surgical peripheral nerve injury and the CIA model of rheumatoid arthritis, microglia in the spinal cord are activated (A). In the case of chemotherapy pain however, circulating monocytes are likely to infiltrate into the spinal cord where they differentiate into macrophages (B). Microglia or macrophages release CatS (C), which cleaves neuronally-expressed fracktalkine (FKN) to produce soluble FKN (sFKN), which then activates CX_3_CR_1_ receptors expressed by microglia and macrophages (D). In turn, microglia and macrophages release pro-nociceptive inflammatory cytokines (E).

Indeed, mechanical hyperalgesia that occurs as a result of intrathecal delivery of either FKN or CatS is prevented by intrathecal administration of a neutralising antibody against FKN [11,42], suggesting that the effects observed as a result of CatS administration are dependent on its FKN-associated actions. Furthermore, whilst intrathecal delivery of CatS results in an increase in the phosphorylation of p38 MAPK in microglia, this effect is inhibited by pre-administration of a FKN neutralising antibody [11]. It is therefore possible that through its actions on FKN, CatS may have indirect effects on microglial responses. Indeed, CatS-induced allodynia is absent in mice that are deficient in the CX_3_CR_1_ [11]. Taken together, this data indicates further that the pro-nociceptive effects of CatS are exerted via FKN signalling. Indeed, following preclinical nerve injury, whilst the total expression level of FKN remains unchanged [18,43], expression of sFKN in the CSF is significantly elevated alongside the increase in CatS activity. Furthermore, in *ex vivo* dorsal horn-with dorsal root attached preparation taken from neuropathic animals, electrical stimulation results in the liberation of FKN only when microglia are activated and in a CatS-dependent manner [36]. CatS-mediated generation of sFKN therefore appears to be pivotal in the underlying mechanisms of pain-like behaviour (Fig. 1).

### 4.2 Peripheral mechanisms

**Fig. 2:**
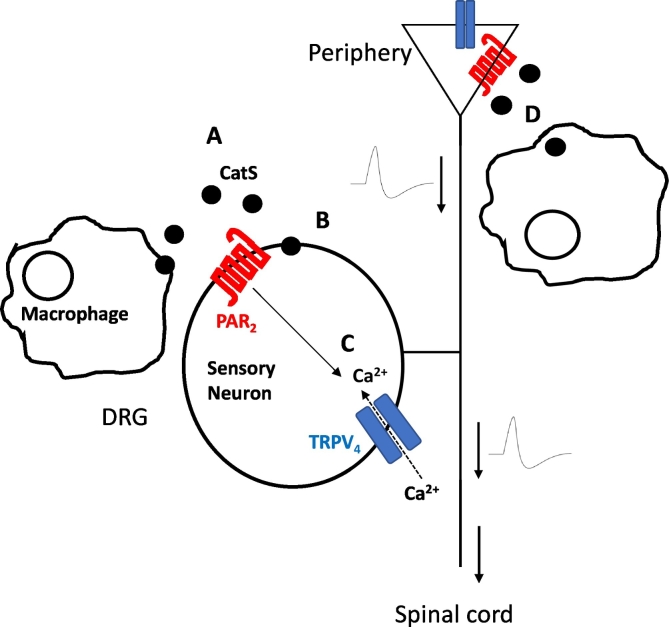
**Schematic representation of pronociceptive CatS-mediated cleavage of PAR**_**2**_. CatS is released from macrophages (A), then cleaves PAR_2_ (B), which in turn activates TRPV_4_ calcium ion channels (C), which mediate nociceptive signalling. (D) Macrophages at the sight of injury also provide a source of CatS, which can target PAR_2_ expressed at nerve terminals [10,48].

Although there is compelling evidence that establishes that the cleavage of FKN is a mechanism by which CatS exerts pro-nociceptive effects centrally, it is important to be aware that it is not the only mechanism by which such effects are achieved and other targets of CatS in the periphery are involved. In addition to FKN, CatS also cleaves protease-activated receptor 2 (PAR_2_). Indeed, intraplantar injection of CatS results in inflammation and hyperalgesia – an effect which is prevented by inhibition of PAR_2_ as well as adenylyl cyclase [44]. This suggests that PAR_2_ activation by CatS also plays an underlying mechanistic role in preclinical pain (Fig. 2). Indeed, one of the effects of CatS cleavage of PAR_2_ that has been observed is the activation of the TRPV_4_ channel [44], which is expressed by sensory neurons and is a well-established transducer of pain signals [45].

In addition to a peripheral role in inflammatory pain, CatS has also been demonstrated to play a role in visceral pain via its effects on PAR_2_ activity. For instance, CatS has been shown to be activated in the colonic mucosa both clinically, in patients with irritable bowel syndrome, and preclinically, in an experimental model of colitis [46]. In the latter, CatS activation has been shown to result in endosomal PAR_2_-dependent hyperexcitability and mechanical allodynia [46].

The presence of more than one mechanism by which CatS inhibition could exert anti-nociceptive effects makes the manipulation of CatS a flexible therapeutic target. Due to the fact that CatS has already received considerable attention in the context of pathological conditions other than chronic pain, CatS inhibitors have already been developed for use in the clinic. Indeed, the CatS inhibitor LY3000328, was found to be well-tolerated in healthy subjects and result in a transient reduction in plasma CatS activity [47]. The development of CatS inhibitors for chronic pain specifically, would therefore be aided by the fact that they are known to be well-tolerated.

## 5 Summary

Attempts to advance our understanding of the underlying mechanisms of chronic pain have resulted in the appreciation for the mechanistic importance of the immune response, in which cysteine peptidases such as Cathepsin S play a variety of roles. In this review we have discussed preclinical evidence for the crucial role of CatS in several models of chronic pain. Pharmacological preclinical studies have revealed that the timing and location of this role are model-dependent. We have also discussed the therapeutic potential of targeting CatS in the context of chronic pain and suggest that in order to most effectively employ CatS inhibitors it would logical to tailor both the delivery route and timing to the nature of injury. Based on the evidence discussed in this review, we tentatively suggest that CatS inhibitors would be most effective if centrally-penetrant, competitive and reversible. In addition, given the crucial role of CatS in antigen presentation, we would expect there to be potential for the use of CatS inhibitors to result in immunosuppression and thus candidate patients should not have compromised immune systems. Nonetheless, CatS provides a potentially useful therapeutic target for the treatment of some chronic pain conditions, whether targeted alone, or as part of combination therapy.

## Conflict of Interest

The authors declare that there are no conflicts of interest

## Funding

Current work In MM lab is supported by the 10.13039/501100000265Medical Research Council UK (MR/M023893/1 and MR/T002883/1), 10.13039/501100012041Versus Arthritis (grant 21961); 10.13039/501100000780European Union's 10.13039/100010661Horizon 2020 research and innovation programme “TOBeATPAIN” under the Marie Skłodowska-Curie grant agreement No 764860.

## Author Contribution

Karli Montague Cardoso and Marzia Malcangio wrote the manuscript and performed literature searches
